# Assessing the contributions of gender, clinical symptoms, and psychometric traits to non-suicidal self-injury behaviors in Chinese adolescents: a nomogram approach

**DOI:** 10.1186/s13034-024-00832-x

**Published:** 2024-11-05

**Authors:** Guanghui Shen, Cheng-Han Li, Qian-Nan Ruan, Su Xu, Wen-Jing Yan

**Affiliations:** 1 Wenzhou Seventh People’s Hospital, Wenzhou, China; 2 Third Affiliated Hospital of Wenzhou Medical University, Wenzhou, China; 3 Department of Psychology, School of Education, Wenzhou University, Wenzhou, China; 4 Wenzhou Key Laboratory of Basic and Translational Research for Mental Disorders, Wenzhou Medical University, Wenzhou, China; 5 Zhejiang Provincial Clinical Research Centre for Mental Illness, Affiliated Kangning Hospital, Wenzhou Medical University, Wenzhou, China

**Keywords:** Adolescents, Non-suicidal self-injury, Mood disorders

## Abstract

**Background:**

Non-suicidal self-injury (NSSI) behaviors among adolescents with mood disorders represent a significant global public health concern. This study aimed to assess the prevalence and identify key predictors of NSSI behaviors in Chinese adolescents diagnosed with depressive or bipolar disorders, addressing a critical gap in the literature.

**Method:**

Data from 2343 participants in the Chinese Adolescent Depression Cohort (CADC) were analyzed. The study employed a comprehensive approach, utilizing medical records, clinical assessments, and validated psychometric instruments. Statistical analyses included chi-square tests, logistic regression, and ROC curve analyses, culminating in the development of a predictive nomogram.

**Results:**

The prevalence of NSSI behaviors was strikingly high at 73.32%. Significant predictors included female gender (AOR = 2.14, 95% CI [1.70, 2.68]), presence of hallucinations (AOR = 1.52, 95% CI [1.18, 1.97]), borderline personality features (AOR = 1.03, 95% CI [1.01, 1.08]), and childhood trauma (AOR = 1.02, 95% CI [1.01, 1.03]). The study’s key contribution is a predictive nomogram with an AUC of 0.74, demonstrating good efficacy in predicting NSSI behaviors.

**Conclusion:**

This research reveals an alarmingly high prevalence of NSSI behaviors in Chinese adolescents with mood disorders and identifies critical predictors spanning demographic, clinical, and psychometric domains. The developed nomogram offers a novel approach for risk assessment, highlighting the importance of comprehensive evaluations in adolescent mental healthcare. These findings have significant implications for developing targeted interventions and improving risk assessment strategies in clinical practice.

**Supplementary Information:**

The online version contains supplementary material available at 10.1186/s13034-024-00832-x.

## Introduction

Non-suicidal self-injury (NSSI) behaviors, particularly among adolescents, have increasingly become a focal point of global public health concern. Characterized by deliberate self-harm without suicidal intent, these behaviors manifest in various forms, including cutting, burning, and hitting oneself. The prevalence rates of NSSI behaviors among adolescents have been reported to be alarmingly high, ranging from 14 to 21% in community samples, indicating its pervasive nature [1, 2]. These behaviors are not only associated with immediate physical harm but also pose a significant risk for future psychopathology, including increased likelihood of suicide attempts [3].

Understanding NSSI behaviors is particularly crucial in certain vulnerable populations, such as adolescents diagnosed with depressive or bipolar disorders. Individuals with mood disorders exhibit a heightened risk for self-injury as compared to the general population [4, 5]. NSSI behaviors in this group are often a byproduct of complex emotional and psychological challenges, including emotional dysregulation and impulsivity, further complicated by the symptoms of their primary mood disorder [6, 7]. Consequently, targeted research in this specific population is imperative for developing effective preventative and interventional strategies.

### Prevalence and risk factors in general populations

Several risk factors for NSSI behaviors have been identified, spanning from individual psychological factors to socio-environmental elements. Psychological factors often implicated include emotional dysregulation, impulsivity, and a history of trauma or abuse [8]. On the socio-environmental front, peer victimization, family dysfunction, and social isolation are commonly associated with self-injury [9, 10]. Importantly, gender differences have also been noted, with females more commonly engaging in self-injury than males [11].

### Prevalence and risk factors in populations with mood disorders

One of the most striking findings in the literature on NSSI behaviors is the elevated prevalence among adolescents with mood disorders. While estimates in general populations are noteworthy, they pale in comparison to the figures observed among those with mood disorders. According to a meta-analysis up to 40% of adolescents with depressive or bipolar disorders have engaged in NSSI behaviors [12, 13]. This represents a nearly twofold increase when compared to community samples, underscoring the severity of the issue in this particular demographic.

A critical aspect of understanding self-injury among adolescents with mood disorders is recognizing the distinct set of risk factors that make this group uniquely susceptible. Emotional dysregulation is often cited as a significant predictor, characterized by intense mood swings, heightened emotional sensitivity, and a limited ability to cope with stressors [14]. Adolescents with depressive disorders may engage in self-injury as a means to manage overwhelming emotional states, while those with bipolar disorders may resort to self-harm during depressive lows to stimulate sensory experiences or during manic highs as a form of risk-taking behavior [13, 15].

Furthermore, the comorbidity of mood disorders with other psychological conditions adds another layer of complexity. For instance, borderline personality disorder features are often prevalent among adolescents with mood disorders and can exacerbate the risk of self-injury [16]. These individuals may experience self-harm as a form of self-punishment or as a means to cope with fears of abandonment or emotional emptiness. Regarding hallucinations specifically, previous research has indicated a relationship between psychotic symptoms and self-harm behaviors in individuals with mood disorders. For example, Dugré et al. [17] found that compliance with self-harm command hallucinations was associated with a history of self-harm in individuals with affective and non-affective psychosis. Additionally, Bornheimer et al. [18] reported associations between hallucinations and suicidal ideation in adults presenting with psychosis. While these studies focused on broader self-harm behaviors, we hypothesized that similar relationships may exist for NSSI specifically.

Lastly, environmental factors should not be overlooked. Adolescents with mood disorders often face stigmatization and social isolation, which can further contribute to emotional distress and the likelihood of self-injury [19]. Additionally, the quality of parent-child relationships and the family environment can significantly impact the prevalence of NSSI behaviors among adolescents with mood disorders [20, 21].

### Problem statement

While the current body of research has provided valuable insights into NSSI behaviors among adolescents, particularly those with mood disorders, several significant gaps remain. One of the most glaring omissions is the lack of comprehensive studies conducted within China or non-Western cultures at large. The majority of the research in this area has been carried out in Western settings, which raises questions about the generalizability of these findings to other cultural contexts [22]. Given that cultural factors can have a significant impact on both the prevalence and the underlying mechanisms of NSSI behaviors, there is an urgent need for studies that explore this phenomenon in diverse cultural landscapes [23, 24].

Additionally, most studies tend to focus on a limited set of variables, often within a single category such as demographic, clinical, or psychometric factors [25, 26]. This narrow scope fails to capture the complex interplay between various types of risk factors, thereby limiting the comprehensiveness of risk assessments and the effectiveness of subsequent intervention strategies. For instance, while there is ample literature on the role of mood disorders or borderline personality features in self-injury [27, 28], less attention has been given to how these clinical factors interact with demographic and psychometric variables. Therefore, our predictor selection was guided by a multidimensional approach, aiming to capture demographic, clinical, and psychometric factors. We included variables that have been consistently associated with NSSI in previous literature (e.g., gender, childhood trauma, borderline personality features) as well as factors that are particularly relevant to our specific population of adolescents with mood disorders (e.g., hallucinations, depressive symptoms).

### The present studied

This study aims to provide an in-depth understanding of NSSI behaviors among Chinese adolescents diagnosed with depressive or bipolar disorders, with specific objectives including assessing prevalence, identifying key demographic and clinical predictors, and evaluating the utility of psychometric measures like borderline personality features and childhood trauma experiences. Based on existing literature and identified research gaps, we hypothesize a high prevalence of NSSI behaviors in this demographic, with significant associations expected between self-injury and variables such as gender, age, presence of hallucinations, and psychometric indicators. Our multidimensional approach seeks to offer a nuanced understanding that can inform targeted intervention strategies, fulfilling a critical gap in existing research.

## Method

### Participants

Data for this study was sourced from the Chinese Adolescent Depression Cohort (CADC), encompassing 2,243 adolescents. The cohort was assembled between December 2020 and December 2021. Recruitment spanned nine provinces and involved 14 medical facilities, including outpatient psychiatric clinics and inpatient wards. Eligibility criteria for study inclusion required participants to be between the ages of 12 and 18, possess a minimum of six years of formal education, and meet the Diagnostic and Statistical Manual of Mental Disorders, Fifth Edition (DSM-5) criteria for either depression or bipolar disorder. These diagnoses were confirmed through comprehensive clinical assessments. Written informed consent was obtained from all participants; for those under 18, consent was provided by a parent or guardian.

### Data collection procedure

Demographic information was obtained from admission medical records. Clinical assessments were conducted by board-certified psychiatric specialists. Following favorable assessments, participants completed a survey, overseen by graduate students with specialization in psychology or psychiatry. To ensure uniformity and rigor in the evaluation process, all researchers involved in the study underwent intensive training prior to data collection. Surveys were administered using tablet computers in quiet ward settings, with an average completion time of approximately 30 min.

### Measures

#### Demographic and clinical information

Demographic data collected from participants’ medical records, which included information on gender, age, parental education levels, years of education, and area of residence. Clinical data were also obtained from medical records and included diagnoses of mental health disorders (as per DSM-5 criteria), presence of hallucinations and delusions. All this data were extracted from admission records filled out by psychiatric directors at the time of participants’ enrollment in the study.

#### NSSI behaviors

NSSI behaviors were assessed using the Functional Assessment of Self-Mutilation (FASM)(Lloyd-Richardson et al., [29], specifically its frequency subscale. Participants reported instances of self-harm over the past year [30]. Based on their responses, they were categorized into NSSI (NSSI) or non-NSSI (NO-NSSI) groups. The frequency subscale of FASM was reliably implemented, achieving a Cronbach’s alpha of 0.85 in the present study.

#### Borderline personality features

We used the Borderline Personality Features Scale for Children (BPFS-C) to assess features of borderline personality [31]. This self-report tool is tailored for younger populations and includes various items that measure different facets of borderline personality traits [32]. Participants rate each item using a 5-point Likert scale. Higher cumulative scores indicate more pronounced features of borderline personality. The BPFS-C demonstrated high internal consistency with a Cronbach’s alpha of 0.92.

#### Peer victimization

Peer victimization was assessed using the Peer Victimization Questionnaire (PVQ). This 16-item self-report instrument measures various aspects of victimization [33]. Participants rated each item on a 5-point Likert scale. Higher total scores indicate more severe experiences of victimization. The Cronbach’s alpha for PVQ in this study was 0.92.

#### Perceived social support

The Multidimensional Scale of Perceived Social Support (MSPSS) was used to gauge perceived social support [34]. Participants rated 12 items on a 7-point Likert scale. Both total and subscale scores were calculated by summing up the responses, with higher scores indicating greater perceived social support. The MSPSS displayed excellent internal consistency with a Cronbach’s alpha of 0.92.

#### Perceived stress

We employed a modified 4-item version of the Perceived Stress Scale (PSS) to assess perceived stress over the last month [35]. Participants rated each item on a 5-point Likert scale. Positively worded items were reverse-scored, and the total score was calculated by summing the responses. Higher scores denote higher stress levels.

#### Depressive symptoms

Depressive symptoms were measured using the Patient Health Questionnaire-9 (PHQ-9)(Levis et al., [36, 37]. Participants rated nine items corresponding to the DSM-IV criteria for major depressive disorder on a 4-point Likert scale, with higher scores signifying more severe depressive symptoms. The internal consistency for the PHQ-9 in this study was excellent, with a Cronbach’s alpha of 0.90.

#### Sleep disorders

The Pittsburgh Sleep Quality Index (PSQI) was utilized to evaluate sleep quality and disturbances over the past month [38]. Participants responded to 19 items that are grouped into seven distinct components, each rated on a scale from 0 to 3. Higher total scores signify poorer sleep quality. In the present study, the PSQI exhibited moderate internal consistency with a Cronbach’s alpha of 0.75.

#### Alexithymia

Alexithymia was assessed using the Toronto Alexithymia Scale (TAS-20). This 20-item self-report instrument measures three core components of alexithymia: difficulty identifying feelings, difficulty describing feelings, and externally oriented thinking [39]. Participants rated each item on a 5-point Likert scale. Higher total scores denote higher levels of alexithymia.

#### Childhood trauma

Childhood trauma using the 28-item Childhood Trauma Questionnaire (CTQ), a widely-validated self-report instrument. The CTQ features items divided into different subscales that capture emotional, physical, and sexual abuse, as well as emotional and physical neglect [40]. Aggregate scores provide insights into the type and severity of experienced trauma. The internal consistency of the CTQ in this study was good, evidenced by a Cronbach’s alpha of 0.88.

### Statistical analysis

Descriptive statistics, comprising means, standard deviations (*SD*), frequencies, and percentages, were calculated for all study variables. Associations between categorical variables, including gender, history of mental disorder diagnosis, hallucinations, and delusions, with the binary outcome of self-injury were assessed using Chi-square tests for independence. Independent samples t-tests were deployed to assess mean differences in continuous variables between NSSI and No-NSSI.

Binary logistic regression was employed to ascertain the relationship between several predictor variables and the propensity of exhibiting NSSI behavior. Initial calculations produced crude odds ratios, quantifying the association of each predictor with NSSI after accounting for demographic variables. Subsequently, adjusted odds ratios were computed to highlight the independent effects of each predictor, considering and controlling for potential confounding variables. This enabled determination of adjusted odds ratios, delineating the independent effects of each variable after accounting for confounders.

The predictive accuracy of risk factors, found significant in the logistic regression, was evaluated using the Receiver Operating Characteristic (ROC) curve analysis. An ROC curve illustrates the sensitivity versus 1-specificity for every potential cutoff. The area under the ROC curve (AUC) acts as a metric of discriminative capability; a larger AUC suggests superior predictive validity. The analysis also pinpointed optimal cutoff points, balancing sensitivity and specificity optimally. By establishing an optimal cutoff value from the ROC curve for a specific questionnaire or test, clinicians can have a tangible reference point.

For predictive modeling, the most influential predictors were amalgamated into a risk prediction nomogram. Discrimination was determined by calculating the area under the curve (AUC) of the receiver operating characteristic curve. The model’s calibration was evaluated by comparing predicted values with observed results, visualized by a calibration curve plot using a 1000 bootstrap resampling procedure. Additionally, Decision Curve Analysis (DCA) and Clinical Impact Curves (CIC) were utilized to gauge the clinical utility of the nomogram across diverse threshold probabilities. Clinicians can use this tool to estimate an individual’s risk based on multiple predictors without dealing with complex calculations.

All statistical procedures were conducted using SPSS version 26 and R version 4.2.1. All tests were two-tailed, and a p-value threshold of less than 0.05 was considered statistically significant.

## Result

### Descriptive analysis

The sample comprised 2343 participants, predominantly diagnosed with Depressive Disorder (84.76%, *N =* 1986). A smaller proportion was diagnosed with bipolar disorder (15.24%, *N =* 357). Concerning familial mental health history, 90.52% (*N =* 2121) had no reported family history of mental disorders, in contrast to 9.48% (*N =* 222) who did. The participants had a mean age of 14.99 years (*SD =* 1.65) and were predominantly female (77.93%, *N =* 1826), with males making up 22.07% (*N =* 517). On average, participants had 9.17 years of schooling (*SD =* 1.76). Various psychological metrics were assessed: the mean score for perceived social support was 47.07 (*SD =* 16.85), and the mean perceived stress score was 14.76 (*SD =* 3.40). (For additional details, see Table 1).

Chi-square tests revealed statistically significant associations between NSSI behaviors and clinical variables. Gender was significantly associated with NSSI (*p <* 0.001), with females being more likely to engage in NSSI (77.82%) compared to males (57.45%). Similarly, a history of mental disorder diagnosis was significantly related to NSSI (*p <* 0.001). Noteworthy associations were also observed between NSSI and reported experiences of hallucinations (*p <* 0.001) and delusions (*χ²* = 33.46, *p <* 0.001) (For additional details, see Supplementary Table 1).

Independent samples *t* test was used to test for differences between No-NSSI and NSSI on psychological characteristics, significant differences were found across all measured variables. Those engaged in NSSI scored significantly lower on perceived social support (*p* < 0.001). Conversely, higher scores were noted on measures of perceived stress, anxiety, and depression (*p* < 0.001). Elevated scores on sleep disorders and borderline personality (*p* < 0.001), were also observed among those who self-injured. Alexithymia, childhood trauma and peer victimization (*p* < 0.001) further distinguished this group (For additional details, see Supplementary Table 2).

**Table 1:** Demographic and clinical characteristics of participants

Variables	*N*	%	M	SD
Diagnose				
Depressive disorder	1986	84.76		
Bipolar disorder	357	15.24		
Family history of mental disorders				
No	2121	90.52		
Yes	222	9.48		
Age			14.99	1.65
Gender				
Male	517	22.07		
Female	1826	77.93		
Education years			9.17	1.76
Location				
Urban	1580	67.43		
Rural	763	32.57		
Education level - Father				
Primary school or below	301	12.85		
Junior high school	882	37.64		
High school	527	22.49		
College or above	633	27.01		
Education level - Mother				
Primary school or below	532	22.70		
Junior high school	809	34.53		
High school	453	19.33		
College or above	549	23.43		
Physical disease				
Yes	65	2.77		
No	2278	97.23		
Previously diagnosed mental disorder				
No	1958	83.56		
Yes	385	16.43		
Hallucination				
No	1471	62.78		
Yes	872	37.22		
Delusion				
No	1472	62.83		
Yes	871	37.17		
Perceived social support			47.07	16.85
Perceived Stress			14.76	3.40
Depression			16.86	7.17
Sleep disorder			12.59	3.68
Borderline personality			81.22	17.81
Alexithymia			67.84	10.57
Childhood trauma			50.58	13.37
Peer victimization			11.35	9.39
NSSI				
No	625	26.68		
Yes	1718	73.32		

### Logistic regression and ROC analysis

The logistic regression analysis was conducted to identify variables that are independently associated with NSSI behaviors among adolescents diagnosed with either depressive or bipolar disorders. This analysis adjusted for various demographic and clinical covariates.

Initially, Crude Odds Ratios (COR) were calculated to evaluate the unadjusted effects of each variable, considering only demographic factors. Gender emerged as a potent demographic predictor; females had 2.38 times greater odds of engaging in NSSI compared to males, 95% CI [1.93, 2.93], *p <* 0.001. Age also had a COR of 0.84, *p* = 0.002, suggesting that younger participants were at higher risk. Clinically, the presence of hallucinations and delusions yielded CORs of 2.21, *p <* 0.001, and 1.67, *p <* 0.001, respectively. Psychometric variables like childhood trauma (COR = 1.04, *p <* 0.001), borderline personality (COR = 1.01, *p <* 0.001), and peer victimization (COR = 1.05, *p <* 0.001) also displayed significance (see Table 2 for further details).

**Table 2:** Logistic regression analysis predicting NSSI among depressed patients

	COR	95%CI	*P*	AOR	95%CI	*P*
Demography factors						
Age	0.84	[0.75, 0.94]	0.002	0.91	[0.81,1.03]	0.13
Gender						
Male	Ref	(--)	(--)	Ref	(--)	(--)
Female	2.38	[1.93, 2.93]	< 0.001	2.14	[1.70, 2.68]	< 0.001
Educational years	0.98	[0.89, 1.09]	0.74	0.96	[0.86, 1.07]	0.4
Location						
Urban	Ref	(--)	(--)	Ref	(--)	(--)
Rural	1.15	[0.91, 1.45]	0.24	1.06	[0.83, 1.36]	0.64
Education level - Father						
Primary school or below	Ref	(--)	(--)	Ref	(--)	(--)
Junior high school	0.92	[0.66, 1.27]	0.62	0.93	[0.66, 1.31]	0.68
High school	0.81	[0.56, 1.18]	0.28	0.80	[0.54, 1.18]	0.26
College or above	0.84	[0.56, 1.18]	0.42	0.86	[0.56, 1.33]	0.51
Education level - Mother						
Primary school or below	Ref	(--)	(--)	Ref	(--)	(--)
Junior high school	1.01	[0.77, 1.33]	0.93	0.96	[0.71, 1.28]	0.78
High school	1.02	[0.73, 1.43]	0.92	1.04	[0.73, 1.50]	0.82
College or above	1.04	[0.71, 1.51]	0.84	1.06	[0.71, 1.57]	0.80
Clinical Factors						
Diagnose						
Depressive disorder	Ref	(--)	(--)	Ref	(--)	(--)
Bipolar sexiness disorder	1.14	[0.88, 1.49]	0.33	1.00	[0.75, 1.34]	0.99
Family history of mental disorders						
No	Ref	(--)	(--)	Ref	(--)	(--)
Yes	0.97	[0.71, 1.34]	0.85	0.79	[0.56, 1.12]	0.18
Physical disease						
Yes	Ref	(--)	(--)	Ref	(--)	(--)
No	0.86	[0.47, 1.50]	0.61	0.97	[0.50, 1.80]	0.93
Previously diagnosed mental disorder						
No	Ref	(--)	(--)	Ref	(--)	(--)
Yes	1.59	[1.21, 2.11]	< 0.001	1.26	[0.94, 1.70]	0.13
Hallucination						
No	Ref	(--)	(--)	Ref	(--)	(--)
Yes	2.21	[1.79, 2.75]	< 0.001	1.52	[1.18, 1.97]	0.001
Delusion						
No	Ref	(--)	(--)	Ref	(--)	(--)
Yes	1.67	[1.36, 2.06]	< 0.001	0.94	[0.74, 1.21]	0.66
Psychometric Factors						
Perceived social support	0.98	[0.98, 0.99]	< 0.001	1.01	[1.00, 1.01]	0.18
Perceived Stress	1.14	[1.11, 1.17]	< 0.001	0.99	[0.95, 1.02]	0.52
Depression	1.08	[1.07, 1.10]	< 0.001	1.01	[0.98, 1.03]	0.53
Sleep disorder	1.15	[1.12, 1.19]	< 0.001	1.04	[1.01, 1.08]	0.02
Borderline personality	1.04	[1.04, 1.05]	< 0.001	1.03	[1.01, 1.08]	< 0.001
Alexithymia	1.05	[1.04, 1.06]	< 0.001	1.01	[1.00, 1.02]	0.18
Childhood trauma	1.04	[1.03, 1.05]	< 0.001	1.02	[1.01, 1.03]	0.002
Peer victimization	1.05	[1.03, 1.06]	< 0.001	1.00	[0.99, 1.02]	0.65

*COR* Crude odds ratio, represents the crude association between the exposure and outcome adjusting for demography variables

AOR Adjusted odds ratio, represents the odds Ratio after adjusting for other variables in the logistic regression model

Upon multivariate adjustment, the Adjusted Odds Ratios (AOR) refined these initial findings. Gender maintained its strong predictive power with an AOR of 2.14, 95% CI [1.70, 2.68], *p <* 0.001. Among clinical predictors, hallucinations remained significant (AOR = 1.52, 95% CI [1.18, 1.97], *p <* 0.001), while delusions were no longer statistically significant (*p* > 0.05). Noteworthy psychometric predictors such as childhood trauma (AOR = 1.02, 95% CI [1.01, 1.03], *p <* 0.01) and borderline personality (AOR = 1.04, 95% CI [1.01, 1.08], *p <* 0.001) sustained their significance in the adjusted model. Other variables like perceived social support and stress lost their significance after adjustment.

**Fig. 1:**
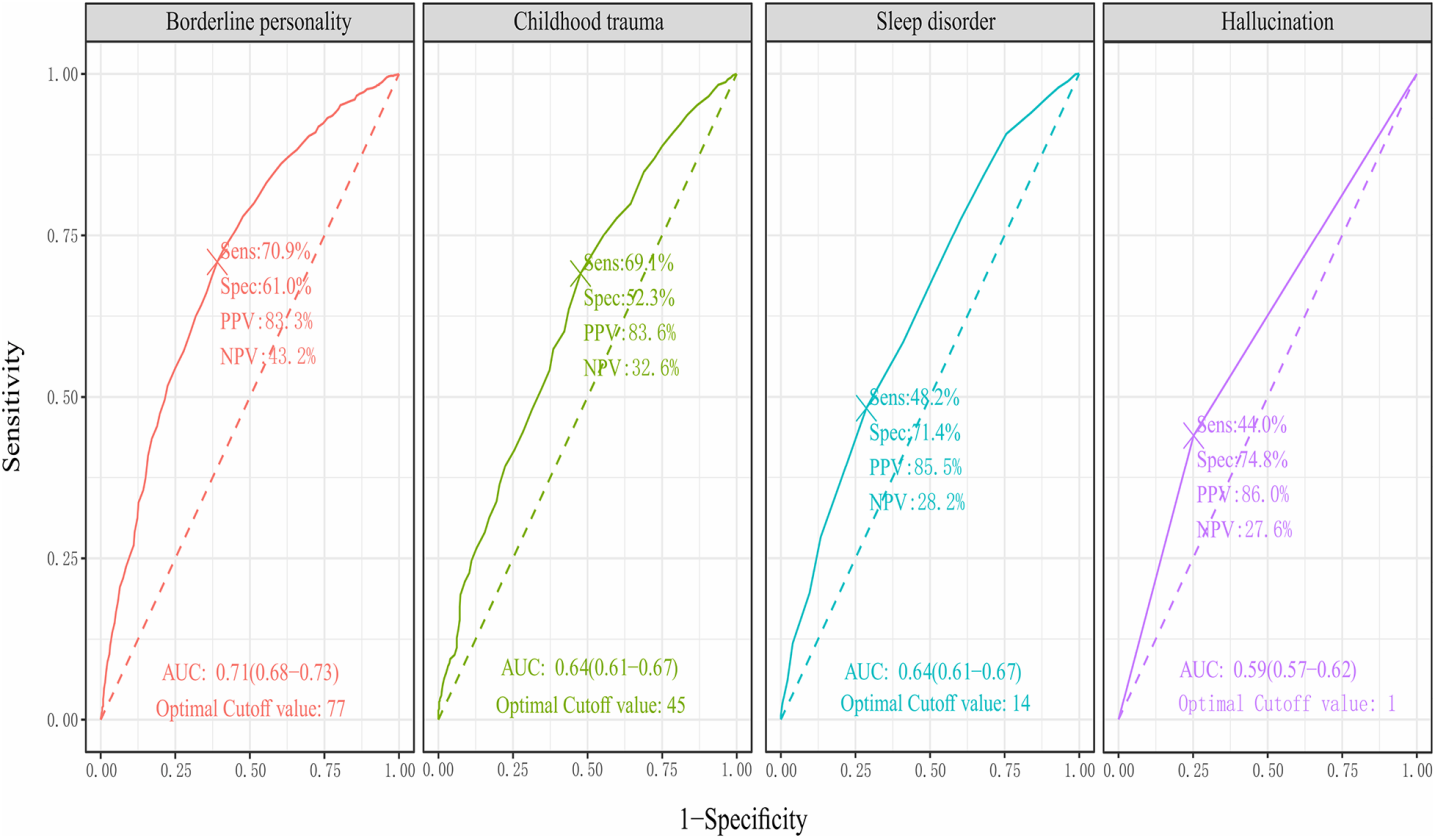
Composite ROC curves for key predictors of NSSI. Sens (Sensitivity): The proportion of actual positive cases which are correctly identified. Spec (Specificity): The proportion of actual negative cases which are correctly identified. PPV (Positive Predictive Value): Among the individuals labeled by the test as positive, the proportion that actually has the condition. NPV (Negative Predictive Value): Among the individuals labeled by the test as negative, the proportion that does not have the condition

The ROC curves were constructed to assess the predictive utility of the identified significant risk factors for NSSI. Borderline personality yielded the highest Area Under the Curve (AUC) of 0.71 (95% CI [0.68, 0.73]), indicating good overall predictive accuracy. An optimal cutoff score was identified at 77, with a sensitivity of 70.9% and a specificity of 61.0%. Childhood trauma also performed moderately well, with an AUC of 0.64 (95% CI [0.61, 0.66]) and an optimal cutoff score of 45 (see Fig. 1 for details). In a stratified analysis by gender, the impacts of risk factors varied between males and females. Remarkably, the borderline personality trait remained a strong predictor for both genders, with an identical AUC of 0.75, indicating strong predictive accuracy. Gender-specific optimal cutoff scores were identified: 81 for males and 75 for females (for a detailed visualization of the ROC curves by gender, refer to Supplementary Figs. 1 and 2).

### Development of the predictive nomogram

#### Construction of nomogram

**Fig. 2:**
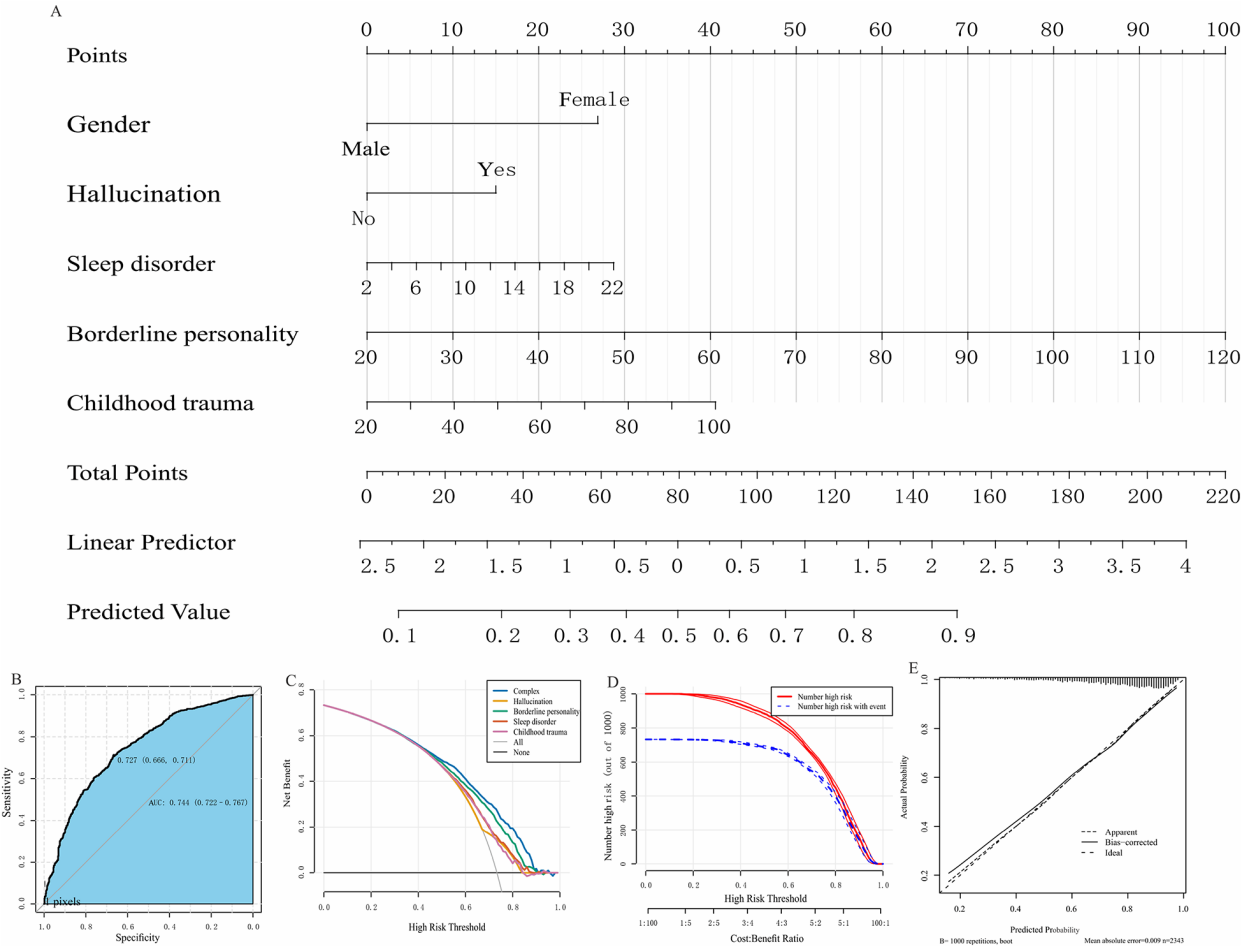
Construction and validation of a nomogram model. **A** Nomogram to predict the occurrence of NSSI. **B** ROC to assess the predictive power of the nomogram model. **C** DCA curve to evaluate the clinical value of the nomogram model. **D** Clinical impact curve based on the DCA curve to assess the nomogram model. **E** Calibration plot a visual tool to assess the agreement between predictions and observations

To facilitate risk assessment for NSSI behavior, we constructed a predictive nomogram based on the optimal predictor variables, as depicted in Fig. 2. In the nomogram, each predictor variable is assigned a score, and a cumulative score is calculated by summing the individual scores of all five variables considered. Figure 2A displays the estimated probabilities corresponding to different cumulative scores; the higher the score, the greater the likelihood of NSSI behavior.

#### Example case

For illustration, consider a female participant who experiences hallucinations, has a sleep disorder score of 16, a borderline personality score of 70, and a childhood trauma score of 60. The scores corresponding to these variables would be approximately 27, 15, 20, 50, and 20, resulting in a total score of 132. According to the nomogram, this score equates to an estimated 80% probability of engaging in NSSI behavior.

#### Nomogram validation

The Area Under the Curve (AUC) for the nomogram was 0.74, with a 95% Confidence Interval (CI) of [0.72, 0.77], as shown in Fig. 2B. Additionally, Decision Curve Analysis (DCA) indicated that using the nomogram for predictions provided more net benefits than employing a single-variable model, specifically at threshold probabilities ranging from 40 to 90% (see Fig. 2C). The calibration plot also revealed good predictive accuracy between the actual probability and predicted probability by bootstrap 1000 (see Fig. 2E).

#### Clinical applicability

The nomogram’s clinical utility was further assessed using Clinical Impact Curves (CIC). The CIC analysis showed that the “high-risk number” curve closely aligned with the “high-risk number with events” curve when the risk threshold was set between 0.75 and 1 (see Fig. 2D). This suggests that the nomogram provides a superior net benefit across practical threshold probabilities, thereby influencing patient outcomes. Importantly, the findings affirm the model’s robust capability to identify cases of NSSI behavior among patients with depression.

## Discussion

The primary aim of this study was to investigate the prevalence and predictors of NSSI behaviors among Chinese adolescents diagnosed with either depressive or bipolar disorders. This research employed a multifaceted approach to data collection and analysis. Key variables were extracted from medical records, and a series of validated psychometric instruments were administered to assess various psychological aspects including, but not limited to, depressive symptoms, borderline personality features, and childhood trauma experiences. The key findings of this study underscore the pervasive prevalence of NSSI behaviors among Chinese adolescents diagnosed with depressive or bipolar disorders, with an alarmingly high rate of 73.32%. Gender and age emerged as significant demographic predictors, corroborating the established understanding that females and younger individuals are at heightened risk. Clinically, hallucinations were a significant risk factor, while psychometric variables like borderline personality features and childhood trauma stood out as robust predictors. These findings are pivotal as they not only extend our understanding of the epidemiology of NSSI behaviors in a non-Western clinical sample but also highlight the necessity for multidimensional risk assessments incorporating demographic, clinical, and psychometric variables for more effective intervention strategies.

This prevalence of NSSI is notably higher than those reported in Western studies. Our data was collected in 2021, which is more recent compared to many oft-cited Western studies. For instance, [41] reported a prevalence of 45% for NSSI among adolescent psychiatric inpatients in the United States, and Groschwitz et al. [42] found a prevalence of 60% among German adolescent psychiatric inpatients. The higher prevalence in our study may reflect a general increasing trend in NSSI behaviors over time, which has been noted in several longitudinal studies (e.g., Wester et al. [43]). Our study focused specifically on adolescents with mood disorders (depressive or bipolar disorders), whereas many previous studies included broader psychiatric populations. Mood disorders are known to be strongly associated with NSSI behaviors [44]. Therefore, our more narrowly defined clinical sample may naturally demonstrate a higher prevalence of NSSI compared to studies with more heterogeneous psychiatric populations.

Our research underscores a significant gender difference in the prevalence of Non-Suicidal Self-Injury (NSSI), corroborating earlier studies [45]. This gender disparity may partially stem from societal norms that condition females to internalize emotions, potentially making NSSI a coping strategy against stress. The adolescent phase, critical for identity formation, may further accentuate females’ vulnerability to NSSI, particularly in response to social conflicts or perceived rejections. It is crucial to note, however, that while males engage in NSSI less frequently, they may employ more lethal methods, as suggested by a previous study [46]. These differences necessitate gender-specific interventions. Our finding of higher NSSI prevalence among females (77.82% vs. 57.45% in males) aligns with Western research. For example, Bresin and Schoenleber [47] in their meta-analysis found that females were 1.5 times more likely to engage in NSSI than males across Western samples.

BPD was identified as a strong predictor of NSSI tendencies, in line with [48]. Individuals with BPD often grapple with emotional dysregulation, including feelings of emptiness and fears of abandonment. These emotions may act as drivers for self-harm, either as a form of emotional release or self-punishment. Given that BPD is often associated with past traumatic experiences, clinicians should assess for underlying personality disorders and traumas when treating NSSI behaviors. Our study showed a strong association between borderline personality features and NSSI (AOR = 1.03, 95% CI [1.01, 1.08]). This aligns with Western research, such as Glenn and Klonsky [49], who found significant correlations between BPD features and NSSI in American adolescents (*r* = 0.52, *p* < 0.001) .

Individuals diagnosed with preexisting mental disorders, particularly mood disorders, displayed an increased susceptibility to NSSI behaviors. These behaviors might serve as coping mechanisms, providing momentary relief from persistent emotional distress [4, 5]. Hallucinations and delusions compound this vulnerability. The distressing nature of these false perceptions can drive individuals toward self-harm as an attempt to mitigate or escape their impact [17] and [18]. offer valuable insights into the role hallucinations and delusions play in self-harm, emphasizing the importance of addressing these symptoms in clinical evaluations.

The intricate relationships among demographic, clinical, and psychometric factors offer a multifaceted perspective on NSSI behaviors, illustrating the issue’s complexity. For example, societal attitudes toward gender roles in China may exacerbate observed gender disparities in NSSI. These cultural norms, which place distinct expectations on males and females, can significantly impact their psychological coping strategies. Existing research corroborates that females, encouraged to internalize emotions, are more prone to NSSI as a form of emotional release [45]. The stigma associated with mental health in China may further compound clinical factors like mood disorders, discouraging individuals from seeking help and increasing the likelihood of self-harm as a coping strategy [19]. Psychometric variables such as borderline personality features and childhood trauma may also interact synergistically, intensifying the risk of NSSI behaviors [48]. Recognizing the interconnectedness of these variables is essential for a holistic approach to intervention, highlighting the importance of multidimensional risk assessments.

The incorporation of a predictive nomogram in our study represents a significant advancement in the field of psychiatric research, particularly in assessing the risk of NSSI behaviors among adolescents with mood disorders. Unlike traditional risk assessment models that often rely on clinician’s subjective judgment or single-variable analysis, our nomogram integrates a comprehensive set of demographic, clinical, and psychometric variables to produce a nuanced and objective risk profile. It demonstrated good predictive accuracy with an Area Under the Curve (AUC) of 0.74, surpassing the threshold generally considered useful for clinical applications. The nomogram’s data-driven approach not only facilitates the early identification of high-risk individuals but also paves the way for more individualized treatment plans and efficient allocation of limited healthcare resources. This methodological innovation aligns with the growing emphasis on data-driven, personalized medicine in psychiatry, offering a practical tool that could be further refined and widely adopted in various clinical settings.

While our study offers meaningful contributions to understanding NSSI behaviors among adolescents with depressive and bipolar disorders in China, several limitations should be acknowledged. First, the study’s geographical focus on China raises questions about the applicability of our findings to other cultural and geographic contexts, a concern emphasized previously [50]. Second, our reliance on medical records for demographic and clinical data introduces the risk of incomplete or inaccurately recorded information. Third, we might missed some important potential variables that may be related to NSSI.

## Conclusion

This study provides valuable insights into the prevalence and risk factors for NSSI behaviors among Chinese adolescents with depressive or bipolar disorders. The alarmingly high prevalence rate of 73.32% underscores the urgent need for targeted interventions in this population. Our findings highlight the complex interplay of demographic, clinical, and psychometric factors in predicting NSSI behaviors. These findings can inform the development of more effective prevention and intervention strategies, ultimately improving mental health outcomes for this vulnerable population.

## Supplementary Information

Below is the link to the electronic supplementary material.

Supplementary file1 (DOCX 762 KB)

## Data Availability

The dataset analyzed in the current study are available in the 360 repository: https://www.yunpan.com/surl_yeDPxWH7ZVW (Code: 1d31).
