# Supplementary material for: Assessing the contributions of gender, clinical symptoms, and psychometric traits to non-suicidal self-injury behaviors in Chinese adolescents: a nomogram approach
Source: Child Adolesc Psychiatry Ment Health. 2024 Nov 5;18:139. doi: 10.1186/s13034-024-00832-x (PMC11536789; doi:10.1186/s13034-024-00832-x)
Supplement: Supplementary file 1 — Supplementary file1 (DOCX 762 KB) [file 13034_2024_832_MOESM1_ESM.docx]

**Supplementary materials**

Table 1 Association Between Demographic and Clinical Factors and NSSI Behavior in Patients with Depressive or Bipolar Disorders

|  | No- NSSI | NSSI | *χ*^2^ | *P* |
| --- | --- | --- | --- | --- |
| Diagnose |  |  | 0.05 | 0.82 |
| Depressive disorder | 532(26.79%) | 1454(73.21%) |  |  |
| Bipolar sexiness disorder | 93(26.05%) | 264(73.95%) |  |  |
| Family history of mental disorders |  |  | 0.01 | 1.00 |
| No | 566(26.69%) | 1555(73.31%) |  |  |
| Yes | 59(26.58%) | 163(73.42%) |  |  |
| Gender |  |  | 84.47 | < 0.001 |
| Male | 220(42.55%) | 297(57.45%) |  |  |
| Female | 405(22.18%) | 1421(77.82%) |  |  |
| Location |  |  | 3.60 | 0.06 |
| Urban | 441(27.91%) | 1139(72.09%) |  |  |
| Rural | 184(24.11%) | 579(75.88%) |  |  |
| Education level - Father |  |  | 2.72 | 0.44 |
| Primary school or below | 75(24.92%) | 226(75.08%) |  |  |
| Junior high school | 223(25.28%) | 659(74.72%) |  |  |
| High school | 148(28.08%) | 379(71.92%%) |  |  |
| College or above | 179(28.28%) | 454(71.72%) |  |  |
| Education level - Mother |  |  | 1.31 | 0.73 |
| Primary school or below | 13(25.94%) | 394(74.06%) |  |  |
| Junior high school | 208(25.71%) | 601(74.29%) |  |  |
| High school | 128(28.26%) | 325(71.74%) |  |  |
| College or above | 151(27.50%) | 398(72.50%) |  |  |
| Physical disease |  |  | 0.06 | 0.81 |
| Yes | 16(24.62%) | 49(75.38%) |  |  |
| No | 609(26.73%) | 1669(73.27%) |  |  |
| Previously diagnosed mental disorder |  |  | 10.91 | < 0.001 |
| No | 549(28.04%) | 1409(71.96%) |  |  |
| Yes | 76(19.74%) | 309(80.26%) |  |  |
| Hallucination |  |  | 75.82 | < 0.001 |
| No | 483(32.83%) | 988(67.17%) |  |  |
| Yes | 142(16.28%) | 730(83.72%) |  |  |
| Delusion |  |  | 33.46 | < 0.001 |
| No | 453(30.77%) | 1019(69.22%) |  |  |
| Yes | 172(19.74%) | 699(80.25%) |  |  |

Table 2 Difference of Psychometric Variables Between with and without NSSI Behavior

|  | No-NSSI | NSSI | *t* | *P* | *Cohen’s d* |
| --- | --- | --- | --- | --- | --- |
| Perceived social support | 50.97 ± 16.81 | 45.65 ± 16.64 | -6.83 | < 0.001 | 0.32 |
| Perceived Stress | 13.57 ± 3.66 | 15.19 ± 3.20 | 10.44 | < 0.001 | 0.49 |
| Depression | 13.64 ± 7.34 | 18.03 ± 6.73 | 13.64 | < 0.001 | 0.64 |
| Sleep disorder | 11.14 ± 3.64 | 13.12 ± 3.55 | 11.84 | < 0.001 | 0.55 |
| Borderline personality | 71.64 ± 18.09 | 84.70 ± 16.38 | 16.95 | < 0.001 | 0.77 |
| Alexithymia | 63.32 ± 11.21 | 69.49 ± 9.82 | 12.94 | < 0.001 | 0.60 |
| Childhood trauma | 46.04 ± 12.38 | 52.23 ± 13.34 | 10.13 | < 0.001 | 0.47 |
| Peer victimization | 8.35 ± 8.40 | 12.45 ± 9.50 | 9.51 | < 0.001 | 0.44 |


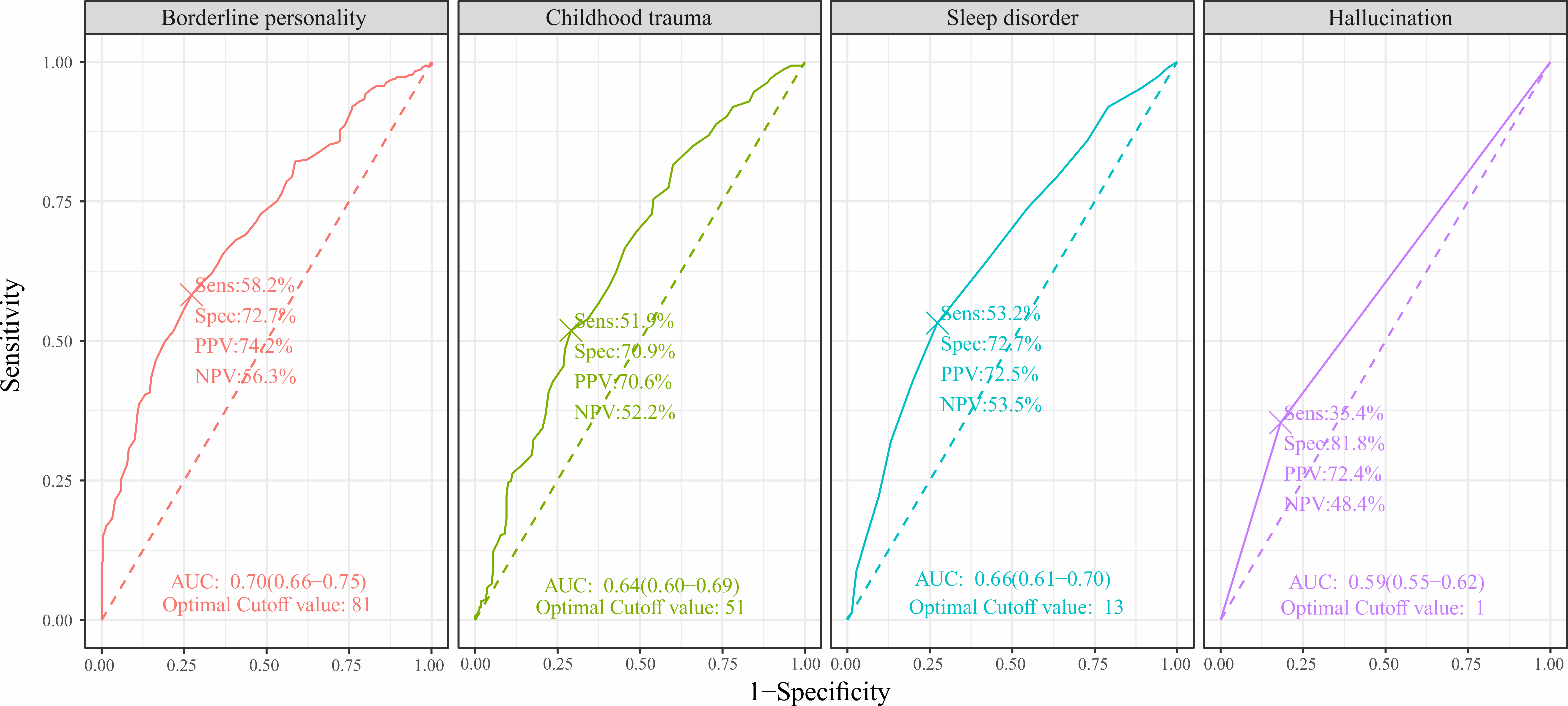


Figure 1. Composite ROC curves for predictors of NSSI in male adolescent patients


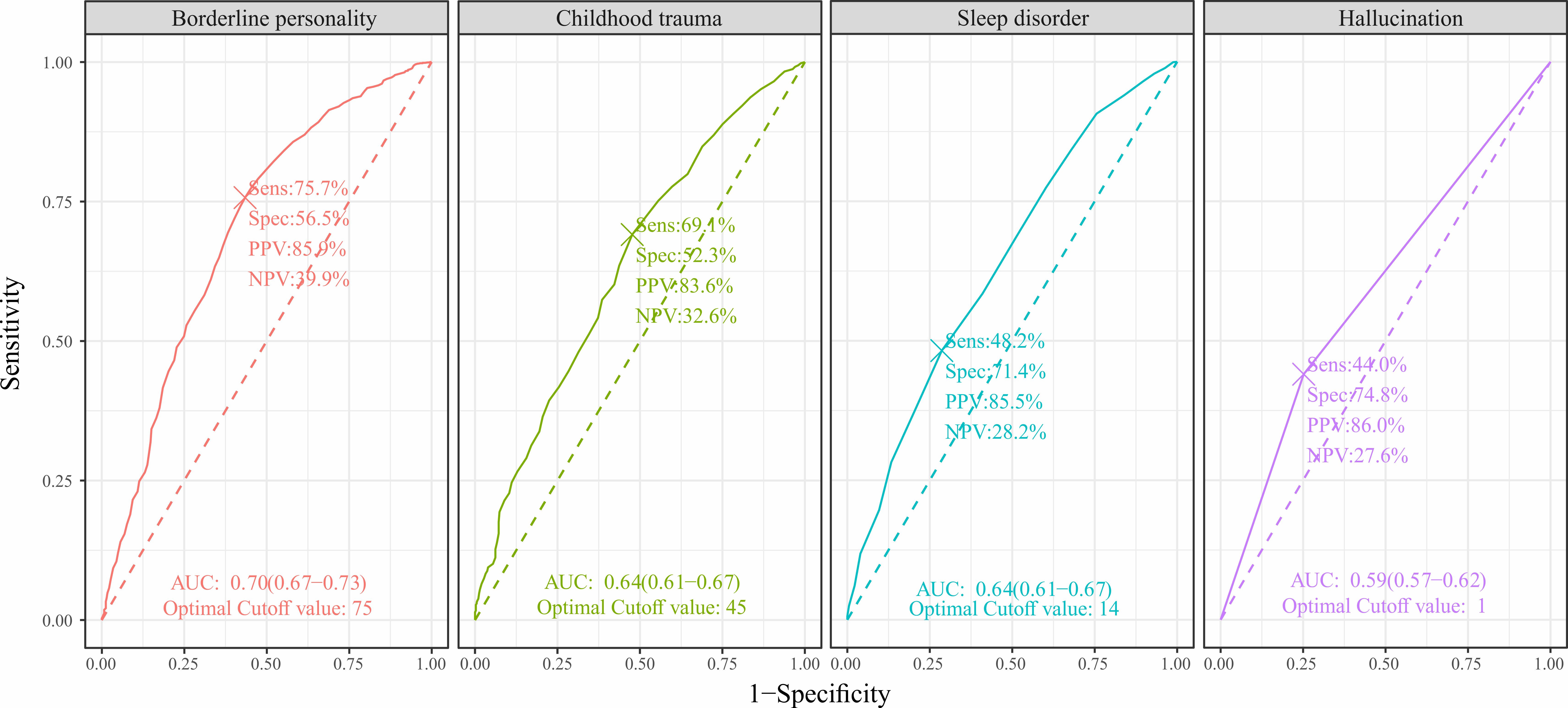


Figure 2. Composite ROC curves for predictors of NSSI in female adolescent patients
